# Determination of Child Waist Circumference Cut Points for Metabolic Risk Based on Acanthosis Nigricans, the Children’s Healthy Living Program

**DOI:** 10.5888/pcd18.210021

**Published:** 2021-06-24

**Authors:** Ashley B. Yamanaka, James D. Davis, Lynne R. Wilkens, Eric L. Hurwitz, Marie K. Fialkowski, Jonathan Deenik, Rachael T. Leon Guerrero, Rachel Novotny

**Affiliations:** 1 Department of Human Nutrition, Food and Animal Science, College of Tropical Agriculture and Human Resources, University of Hawai‘i at Mānoa, Honolulu, Hawai‘i; 2 Department of Biostatistics and Quantitative Health Sciences, John A. Burns School of Medicine, University of Hawai‘i at Mānoa, Honolulu, Hawai‘i; 3 Biostatistics and Informatics Shared Resource, University of Hawai‘i Cancer Center, Honolulu, Hawai‘i; 4 Office of Public Health Studies, Thompson School of Social Work and Public Health, University of Hawai‘i at Mānoa, Honolulu, Hawai‘i; 5 Department of Tropical Plant and Soil Sciences, College of Tropical Agriculture and Human Resources, University of Hawai‘i at Mānoa, Honolulu, Hawai‘i; 6 Office of Research and Sponsored Programs, University of Guam, Mangilao, Guam

## Abstract

**Introduction:**

Waist circumference is a common anthropometric measure for predicting abdominal obesity and insulin resistance. We developed optimal waist circumference cut points for children aged 2 to 8 years in the US-Affiliated Pacific (USAP) region based on the relationship of waist circumference and acanthosis nigricans in this population.

**Methods:**

We conducted a cross-sectional analysis from the Children’s Healthy Living Program’s 2012–2013 data on 4,023 children. We used receiver-operating characteristic analysis to determine the sensitivity and specificity for acanthosis nigricans across waist circumference, by sex and age. We determined optimal waist circumference cutoff points corresponding to Youden index (*J*), (equal to [sensitivity + specificity] – 1), with acanthosis nigricans. We compared these cut points with the 90th percentile.

**Results:**

The 90th-percentile cut points for boys aged 2 to 5 years (58.15 cm) and 6 to 8 years (71.63 cm) were slightly higher than for girls in both age groups (aged 2–5 y, 57.97 cm; 6–8 y: 70.37 cm). The optimal cut points (corresponding to the highest sensitivity and specificity) were as follows: for boys aged 2 to 5 years, 90th percentile (58.25 cm; sensitivity, 48.0%; specificity, 91.5%); for boys aged 6 to 8 years, 78th percentile (63.59 cm; sensitivity, 86.8%; specificity, 82.8%); for girls aged 2 to 5 years, 62nd percentile (53.27 cm; sensitivity, 71.4%; specificity, 63.1%), and for girls aged 6 to 8 years, 80th percentile (63.63 cm; sensitivity, 55.4%; specificity, 82.9%).

**Conclusion:**

Among USAP children, waist circumference was a reasonable predictor for acanthosis nigricans. Further analysis is warranted to examine causes of acanthosis nigricans at lower-than-expected waist circumference percentiles. The cut points can be used for early detection of metabolic risk.

**Summary**

**What is already known on this topic?**

Obesity and insulin resistance are primary risk factors for metabolic syndrome. Many studies have reported waist circumference cut points in association with metabolic risk in children. No widely accepted waist circumference cut points for children exist.

**What is added by this report?**

We conducted a cross-sectional analysis to determine waist circumference cut points to predict acanthosis nigricans, a measure for metabolic syndrome, in children. We found waist circumference cut points lower than the 90th percentile.

**What are the implications for public health practice?**

Waist circumference can be used as a screening tool for early detection of metabolic risk in children.

## Introduction

Many studies have indicated a relationship between adiposity and the risk of chronic diseases early in life. Body mass index (BMI) is a common, widely recognized indicator of adiposity that is used to identify people who are overweight and obese (1,2). However, abdominal adiposity has been found to be more related than overall adiposity to metabolic disease. Unlike waist circumference, BMI does not consider body fat distribution (3). Waist circumference is a commonly used anthropometric measure for abdominal obesity and is an independent predictor of insulin resistance (4). The prevalence of childhood obesity worldwide indicates a need to identify children with metabolic risk for early intervention to prevent and treat metabolic changes associated with chronic diseases, such as through the establishment of waist circumference cut points. The International Diabetes Federation (IDF) suggests the 90th percentile as a waist circumference cut point for children aged 6 years or older (5). No widely accepted waist circumference cut points or reference data set exist for children and/or adolescents, although some country-specific cut points have been suggested (6,7).

Acanthosis nigricans (acanthosis) is a skin disorder characterized by hyperpigmentation, hyperkeratosis, and papillomatosis and appears as a dark velvety thickening, particularly in skin creases (8). Acanthosis is found on the posterior neck, axillae, knees, elbows, and groin. Studies have shown an association between acanthosis and hyperinsulinemia and obesity (9–13). The presence of acanthosis indicates an increased risk for development of type 2 diabetes and may be a noninvasive marker for metabolic changes (14,15). Pediatric endocrinology guidelines state that if acanthosis is detected in a child, further biochemical testing for insulin resistance is not warranted (16). The prevalence of acanthosis is higher among African American, Hispanic, and Native American people and Pacific Islanders than among non-Hispanic White people (17,18). Strong evidence shows a disproportionate prevalence of type 2 diabetes and obesity among racial/ethnic minority populations (19–21).

The population in the US-Affiliated Pacific region (USAP) is a medically underserved racial/ethnic minority population and underrepresented in national comprehensive nutrition and health research studies. The USAP region comprises Hawai‘i and Alaska, the US territories Guam and American Samoa, the US Commonwealth of the Northern Mariana Islands, and the Freely Associated States of Micronesia (consisting of the Federated States of Micronesia [Pohnpei, Chuuk, Yap, and Kosrae], the Republic of the Marshall Islands, and the Republic of Palau). These jurisdictions do not monitor nutrition through such means as the National Health and Nutrition Examination Survey for nutrition-related health promotion (22). Limited data on diet, physical activity, obesity, and other health-related indicators in this region restrict understanding of the care and action needed to control the region’s epidemic of noncommunicable chronic diseases (23). However, the Children’s Healthy Living (CHL) program collected health data from 2012 through 2015 to provide a unique opportunity to fill the knowledge gap in this region (24).

The objective of this study was to develop optimal waist circumference cut points to identify children with metabolic risk in the USAP region based on the relationship of waist circumference and acanthosis in this population. If waist circumference cut points for children were established, early screening for possible health risks associated with abdominal obesity could be helpful in disease prevention.

## Methods

The CHL program is a multisite, multilevel community randomized intervention trial and prevalence study of remote, medically underserved racial/ethnic minority populations in the USAP; it was conducted from October 7, 2012, through October 25, 2015. CHL’s mission was to elevate the capacity of the region to build healthy food and physical environments to prevent childhood obesity (24). The CHL program recruited children aged 2 to 8 years from selected communities to collect health-related information at 1 time point on prevalence in the Freely Associated States of Micronesia and at 2 time points for intervention in the other USAP jurisdictions. This study was a cross-sectional secondary analysis from CHL’s time 1 data (October 2012 through December 2013). Additional information about the CHL program is provided elsewhere (25–27). Written informed consent was obtained from parents or guardians, and assent was obtained from children. The protocol and procedures for this study were approved by the institutional review boards at the University of Hawai‘i at Mānoa, the University of Alaska at Fairbanks, and the University of Guam. Other jurisdictions ceded approval to the University of Hawai‘i.

### Exposures

**Body size measurements.** Measurements of weight, height, and waist circumference were obtained with the use of standardized techniques by 2 trained research team members (28). Zerfas criteria (29,30) were used to standardize the measurements of research team members against the measurements of a certified anthropometrist (R.N.). Waist circumference was measured to the nearest 1 mm by using a calibrated anthropometric tape measure at the umbilicus. Weight was measured to the nearest 0.1 kg by using a calibrated digital scale. Height was measured to the nearest 1 mm by using a calibrated stadiometer. Each measurement was performed in sets of 3 replicates and repeated until the values were consistent (2 values within 2 units); we used the average for analysis.

**Acanthosis assessment.** Each child was examined for the presence or absence of acanthosis on the back of the neck, as a skin indicator of insulin resistance, by trained staff members according to the protocol of Burke et al (31). The severity of acanthosis on the back of the neck, compared with other body sites, is more consistently associated with insulin resistance (31). Acanthosis was rated for severity on a scale of 0 to 4 points, with a score of 0 indicating absence and a score of 1, 2, 3, or 4 indicating presence (1, least severe; 4, most severe). If acanthosis was present, the child’s parent was referred to a health professional.

**Statistical analysis.** We calculated means and percentiles for waist circumference, by age and sex group. BMI was calculated as weight in kg divided by height in meters squared, and BMI percentiles were calculated according to age-specific and sex-specific growth reference curves published by the Centers for Disease Control and Prevention (32). We used sex-specific and sex–age-group–specific receiver-operating characteristic (ROC) analysis to investigate the ability of waist circumference to predict the presence or absence of acanthosis (33). The ROC curve plots sensitivity against value for 1 minus specificity for the identification of acanthosis across the range of waist circumference values. We determined the optimal waist circumference cut point as the waist circumference value that corresponds to the maximum Youden index (*J*), computed as ([sensitivity + specificity] – 1) (34). We then performed binary logistic regression models, adjusting for age and the presence of acanthosis (1–4 vs 0 on the Burke scale), to examine the predictive performance of an indicator variable for waist circumference divided at the optimal cut point among boys and girls separately. A *P* value < .05 was considered significant. We used SAS software version 9.4 (SAS Institute Inc) to perform statistical analyses.

## Results

A total of 4,023 children (2,026 boys and 1,997 girls) aged 2 to 8 years were included in the study. Among boys and girls aged 2 to 5 years, most participants were Native Hawaiian/Other Pacific Islanders (Table 1). Acanthosis was present in about 5% (n = 203) of the population overall, and the prevalence of obesity was higher among boys (15.8%) than girls (10.8%). In general, waist circumference increased with age group among boys and girls (Table 2). Boys had higher waist circumference values than girls at every percentile level except for the 95th percentile for the group aged 2 to 5 years. Values in the 90th percentile (recommended by the IDF as a cut point for risk of diabetes) for boys aged 2 to 5 years (58.15 cm) and 6 to 8 years (71.63 cm) were slightly higher than values for girls in both age groups (2–5 y, 57.97 cm; 6–8 y, 70.37 cm).

**Table 1:** Characteristics of Boys and Girls Aged 2 to 8 Years Participating in the Children’s Healthy Living Program, US-Affiliated Pacific Region, 2012–2013

Characteristics	No. (%)		
	Boys	Girls	Total
**Total**	2,026 (50.4)	1,997 (49.6)	4,023 (100.0)
**Age group, y**			
2–5	1,264 (62.4)	1,243 (62.2)	2,507 (62.3)
6–8	762 (37.6)	754 (37.8)	1,516 (37.7)
**Race/ethnicity**			
American Indian/Alaska Native	17 (0.8)	19 (0.9)	36 (0.9)
Asian	149 (7.4)	132 (6.6)	281 (6.9)
Black	4 (0.2)	4 (0.2)	8 (0.2)
≥1 Race	383 (18.9)	399 (20.0)	782 (19.4)
Native Hawaiian/Other Pacific Islander	1,337 (66.0)	1,342 (67.2)	2,679 (66.6)
White	125 (6.2)	96 (4.8)	221 (5.5)
Missing	11 (0.5)	5 (0.3)	16 (0.4)
**Jurisdiction**			
Palau	97 (4.8)	85 (4.3)	182 (4.5)
Yap	89 (4.4)	101 (5.1)	190 (4.7)
Guam	359 (17.7)	349 (17.5)	708 (17.6)
Commonwealth of the Northern Mariana Islands	307 (15.2)	295 (14.8)	602 (15.0)
Chuuk	103 (5.1)	85 (4.3)	188 (4.7)
Pohnpei	88 (4.3)	88 (4.4)	176 (4.4)
Kosrae	103 (5.1)	84 (4.2)	187 (4.7)
Republic of the Marshall Islands	87 (4.3)	125 (6.3)	212 (5.3)
American Samoa	328 (16.2)	307 (15.4)	635 (15.8)
Hawai‘i	287 (14.2)	335 (16.8)	622 (15.5)
Alaska	178 (8.8)	143 (7.2)	321 (8.0)
**Acanthosis nigricans**			
Yes	101 (5.0)	102 (5.1)	203 (5.0)
No	1,918 (94.7)	1,886 (94.4)	3,804 (94.6)
Missing	7 (0.4)	9 (0.5)	16 (0.4)
**Body mass index^a^**			
Underweight (<5th percentile)	55 (2.7)	54 (2.7)	109 (2.7)
Healthy weight (5th–85th percentile)	1,389 (68.6)	1,454 (72.8)	2,843 (70.7)
Overweight (85th–95th percentile)	261 (12.9)	272 (13.6)	533 (13.3)
Obese (≥95th percentile)	319 (15.8)	216 (10.8)	535 (13.3)
Missing	2 (0.1)	1 (0.1)	3 (0.1)

a Cut points defined in Kuczmarski et al (32).

**Table 2:** Waist Circumference (cm) by Percentile^a^ Among Boys and Girls Aged 2 to 8 Years Participating in the Children’s Healthy Living Program, US-Affiliated Pacific Region, 2012–2013

Age, y	n	Percentile								
		5th	10th	15th	25th	50th	75th	85th	90th	95th
**Overall**	4,023	46.93	48.27	49.23	50.53	53.30	57.17	60.13	63.57	70.27
**Boys**										
**Overall**	2,026	47.33	48.47	49.33	50.67	53.43	57.33	60.30	64.03	71.07
2–5	1,264	46.60	47.30	48.50	49.73	52.07	54.80	56.70	58.15	61.20
6–8	762	49.47	50.57	51.67	53.30	56.57	62.10	67.67	71.63	78.67
**Girls**										
**Overall**	1,997	46.50	48.00	49.10	50.37	53.23	56.90	59.97	64.03	71.07
2–5	1,243	45.83	47.10	48.03	49.53	51.97	54.63	56.30	57.97	61.40
6–8	754	49.07	50.13	51.03	52.57	56.17	60.90	66.20	70.37	75.93

a Waist circumference percentile: calculated from the sample’s waist circumference normality distribution, by sex and age group. To convert centimeters to inches, multiply centimeters by 0.39.

The optimal waist circumference cut points for predicting acanthosis among all children aged 2 to 8, determined by using the Youden index, were equivalent to the 85th percentile for both sexes (Table 3). The sex–age-group–specific waist circumference cut points were, for boys, at the 90th (58.25 cm, aged 2–5 y) and 78th (63.59 cm, aged 6–8 y) percentiles, and for girls, at the 62nd (53.27 cm, aged 2–5 y) and 80th (63.63 cm, aged 6–8 y) percentiles. These waist circumference cut points represent an increased likelihood of metabolic risk, based on the presence of acanthosis. At the optimal cut point for waist circumference, 48.0% to 86.8% (sensitivity) of children were correctly classified as having acanthosis and 63.1% to 91.5% (specificity) of children were correctly classified as not having acanthosis. However, when we used IDF criteria, sensitivity was lower (37.5%–60.4%) and specificity was higher (90.8%–93.3%) than optimal cut point values. The areas under the ROC curves differed between the optimal sex-specific and sex–age-group–specific and IDF criteria values.

**Table 3:** Sex-Specific and Sex–Age-Group–Specific Optimal Waist Circumference Cut Point Values for Acanthosis Nigricans Among Boys (n = 2,026) and Girls (n = 1,997) Aged 2 to 8 Years Participating in the Children’s Healthy Living Program, US-Affiliated Pacific Region, 2012–2013

Age, y	Percentile	Value, cm^a^	Area Under the Curve (95% CI)	Sensitivity, %	Specificity, %	Youden *J* b	Positive Predictive Value	Negative Predictive Value
**Boys**								
**Overall**	85th	60.12	0.77 (0.71–0.83)	64.1	86.9	0.51	0.21	0.98
2–5	90th	58.25	0.69 (0.60–0.78)	48.0	91.5	0.40	0.19	0.98
6–8	78th	63.59	0.86 (0.79–0.92)	86.8	82.8	0.70	0.27	0.99
**By International Diabetes Federation criteria^c^**								
**Overall**	90th	63.57	0.73 (0.68–0.78)	58.2	91.9	0.50	0.28	0.98
2–5	90th	58.15	0.69 (0.63–0.77)	48.0	91.0	0.39	0.18	0.98
6–8	90th	71.63	0.76 (0.69–0.83)	60.4	93.3	0.54	0.41	0.97
**Girls**								
**Overall**	85th	59.57	0.72 (0.66–0.77)	50.5	85.8	0.36	0.16	0.97
2–5	62nd	53.27	0.72 (0.64–0.79)	71.4	63.1	0.35	0.07	0.98
6–8	80th	63.63	0.70 (0.62–0.78)	55.4	82.9	0.38	0.21	0.96
**By International Diabetes Federation criteria^c^**								
**Overall**	90th	64.03	0.64 (0.60–0.69)	39.0	92.6	0.32	0.23	0.96
2–5	90th	57.97	0.64 (0.58–0.72)	38.8	90.8	0.30	0.15	0.97
6–8	90th	70.37	0.64 (0.58–0.70)	37.5	91.8	0.29	0.27	0.95

a To convert centimeters to inches, multiply centimeters by 0.39.

b The optimal cutoff value providing the best tradeoff between sensitivity and specificity.

c Zimmet et al (5).

## Discussion

This study is the first to provide age group–specific and sex-specific waist circumference cut points for children, predominantly Native Hawaiian/Other Pacific Islanders aged 2 to 8 years, in the USAP region, based on the presence of acanthosis, an indicator of metabolic syndrome. Limited data exist on ideal or acceptable waist circumference cut points for identifying the risk of metabolic syndrome among young children. However, an increasing number of studies support the use of waist circumference instead of BMI to readily identify children with insulin resistance or metabolic syndrome in clinical settings (35,36). Derived waist circumference cut points for children to identify metabolic syndrome or cardiovascular risk factors have been suggested in the US and other countries (37–40). A US study on children and adolescents identified waist circumference cut points for boys at the 94th percentile and for girls at the 84th percentile in association with cardiometabolic risk (38). A study of Chinese school-aged children reported the 90th percentile for boys and the 84th percentile for girls as waist circumference cut points for predicting cardiovascular disease risk factors (40). These studies suggest a need for additional data to develop and validate appropriate cut points for racially/ethnically diverse Pacific Island populations, which may differ from the cut points for other populations, such as non-Hispanic White people, by age and sex, and help interpret health implications in relation to body size.

Our study used ROC analysis to evaluate the optimal cut point value of waist circumference to predict the presence of acanthosis. The derived waist circumference cut points had a low sensitivity for boys aged 2 to 5 years and girls aged 6 to 8 years, and only 5% of the population were identified as having acanthosis. Using separate cut points for children aged 2 to 5 and children aged 6 to 8 years predicted acanthosis better than a single cut point for the entire age range. The age group–specific percentiles identified as the optimal cut point for boys and girls, except boys aged 2 to 5 years, were lower than the IDF recommendations for diagnosing metabolic syndrome among children aged 6 years or older (5). IDF’s criteria do not identify waist circumference cut points for children younger than 6 years; they also do not consider differences in waist circumference among racial/ethnic groups that may have additional metabolic risk. A single value for waist circumference cut points across sexes and racial/ethnic groups may be easier to apply but is less sensitive in identifying children with metabolic risk factors (5). In 2004, the World Health Organization recommended lower BMI cut points for the Asian population based on evidence that demonstrated increased risk of cardiometabolic disorders at lower BMI levels compared with other racial/ethnic groups (41).

Our study had several strengths and limitations. The strengths were the novel method used to determine waist circumference cut points for children living in the USAP region and the large sample size across multiple jurisdictions. The study is representative of Native Hawaiian/Other Pacific Islander children (26). In addition, the CHL program’s rigorous anthropometric methodology for standardizing measures decreases measurement error (28). Scientific consensus is needed on the anatomical measurement site for young children and acceptable levels of error in measurement in further development of methods using waist circumference measures. A limitation was that the study did not account for other measurements related to metabolic syndrome such as blood pressure, triglyceride levels, or cholesterol; these measurements were not collected in the CHL program. In addition, the cross-sectional design does not allow for temporal consideration of waist circumference for acanthosis risk. Lastly, the CHL study sampled communities with a high percentage of indigenous populations and may not have been a representation of the overall jurisdiction. Urban communities in Hawai‘i and rural villages in Alaska were not included. 

The USAP region is undergoing a nutrition and epidemiologic transition, a rapid shift in diet and physical activity, caused by environmental changes and an increase in wealth (42). In addition, colonialism led to changes in indigenous cultural practices, traditional diets, foods, sovereignty, customs, and identity (43). The indigenous people of the USAP region have experienced a concomitant trend of weight gain, obesity, and behavior change. The level of central adiposity among children in the USAP region is a major public health concern because overweight and obesity may lead to chronic noncommunicable diseases. Our study suggests a need for lower percentile cut points for children in the USAP region, who may be at greater risk than children of other races/ethnicities for metabolic diseases such as diabetes. The use of waist circumference measurements is recommended to define health risks for policy development and intervention strategies. Early detection and screening of waist circumference among children can lead to prevention-oriented research and practice to decrease the likelihood of adverse health outcomes later in life among children with metabolic risk.

Our study provides waist circumference values of children aged 2 to 8 years in the USAP region that represent optimal age group–specific and sex-specific cut points to predict the presence of acanthosis. These results add to available reference values and serve as an additional tool in screening for central adiposity and metabolic risk in young children. Currently, no gold standard or cut points for degree of adiposity in children exist for predicting the risk of metabolic syndrome throughout the life span. The derived waist circumference cut points described in our study provide guidelines for evaluating waist circumference in an epidemiologic setting in the USAP region. Further studies should examine the interaction of BMI and waist circumference with acanthosis and other chronic disease risk factors for metabolic syndrome. Our findings were based on a cross-sectional design and need to be validated in a longitudinal study and studies in additional populations.
